# Histological Alterations and Interferon-Gamma and AKT-mTOR Expression in an Experimental Model of Achilles Tendinopathy—A Comparison of Stem Cell and Amniotic Membrane Treatment

**DOI:** 10.3390/biomedicines13020525

**Published:** 2025-02-19

**Authors:** Guilherme Vieira Cavalcante, Rosangela Fedato, Lucia de Noronha, Seigo Nagashima, Ana Paula Camargo Martins, Márcia Olandoski, Ricardo Pinho, Aline Takejima, Rossana Simeoni, Julio Cesar Francisco, Luiz César Guarita-Souza

**Affiliations:** 1 Postgraduate Program in Health Sciences, Department of Operative Technique and Experimental Surgery, Experimental Laboratory of Institute of Biological and Health Sciences, School of Medicine and Life Sciences, Pontifical Catholic University of Paraná (PUCPR), Curitiba 80215-901, Brazil; guaritasouzalc@hotmail.com; 2 Postgraduate Program in Health Sciences, Experimental Laboratory of Institute of Biological and Health Sciences, School of Medicine and Life Sciences, Pontifical Catholic University of Paraná (PUCPR), Curitiba 80215-901, Brazil; rofedato@yahoo.com.br; 3 Postgraduate Program in Health Sciences, Department of Medical Pathology, School of Medicine and Life Sciences, Pontifical Catholic University of Paraná (PUCPR), Curitiba 80215-901, Brazil; lnno.noronha@gmail.com (L.d.N.); seigocap@gmail.com (S.N.); ana.martins@pucpr.br (A.P.C.M.); 4 Department of Biostatics, School of Medicine and Life Sciences, Pontifical Catholic University of Paraná (PUCPR), Curitiba 80215-901, Brazil; marcia.olandoski@pucpr.br; 5 Experimental Laboratory of Institute of Biological and Health Sciences, Pontifical Catholic University of Paraná (PUCPR), Curitiba 80215-901, Brazil; ricardo.pinho@pucpr.br (R.P.); alinetakejima@yahoo.com.br (A.T.); rsimeoni@ig.com.br (R.S.); julio.apfr@gmail.com (J.C.F.)

**Keywords:** tendinopathy, Achilles tendon, mononuclear stem cells, amniotic membrane, interferon-γ, AKT-mTOR

## Abstract

Achilles tendon injuries are extremely common and have a significant impact on the physical and mental health of individuals. Both conservative and surgical treatments have unsatisfactory results. The search for new therapeutic tools, using cell therapies with stem cells (SC) and biological tissues, such as amniotic membranes (AM), has proved useful for the regeneration of injured tendons. **Background/Objectives:** This research was carried out to assess the capacity of tissue repair in animal models of Achilles tendinopathy, in which rats were submitted to complete sections of the tendon, and the effects of using bone marrow SC and/or AM graft are evaluated. **Methods:** Thirty-seven Wistar rats, submitted to complete surgical section of the Achilles tendon and subsequent tenorrhaphy, were randomized into four groups: Control Group (CG), received saline solution; SC Group (SCG) received an injection of SC infiltrated directly into the tendon; AM Group (AMG), the tendon was covered with an AM graft; SC + AM Group (SC+AMG), has been treated with an AM graft and SC local injection. Six weeks later, the Achilles tendons were evaluated using a histological score and immunohistochemical pro-healing markers such as Interferon-γ, AKT, and mTOR. **Results:** There were no differences between morphometric histological when evaluating the Achilles tendons of the samples. No significant differences were found regarding the expression of AKT-2 and mTOR markers between the study groups. The main finding was the presence of a higher concentration of Interferon-γ in the group treated with SC and AM. **Conclusions:** The isolated use of SC, AM, or the combination of SC-AM did not produce significant changes in tendon healing when the histological score was evaluated. Similarly, no difference was observed in the expression of AKT-2 and mTOR markers. An increase in the expression of Interferon-γ was observed in SC+AMG. This suggests that such therapies may be potentially beneficial for the regeneration of injured tendons. However, as tendon repair mechanisms are very complex, further studies should be carried out to verify the benefits of the tendon structure and function.

## 1. Introduction

Extremely common in clinical practice, tendon disorders are an important cause of morbidity and result in pain, reduced mobility, and inability to practice physical activities [1]. Anatomically, tendons are structures that connect muscles to bones, transmitting mechanical forces and ensuring movement, which is why they are particularly susceptible to stress injuries [2]. Due to its low vascularization, tendons have limited healing potential, both in acute and chronic injuries. In this context, Achilles tendinopathies are of great importance, characterized by their wide prevalence, and are a clinical condition that leads to significant pain and disability [3,4,5].

Achilles tendons without structural alterations have great tensile strength, which is due to their composition of around 90% linearly arranged type I collagen fibers. However, in the event of injury, the scarring process inherent to the tissue occurs through the formation of a fibrous scar, which remains for around 10 weeks and has type III collagen as its main component. This process is characterized by the random arrangement of fibers, which have different mechanical and biochemical properties, such as elasticity and resistance, to those of the intact tendon, resulting in greater fragility and its clinical repercussions [1,2]. 

The therapeutic approach to Achilles tendinopathy is centered on two main axes: conservative treatment or surgical treatment. Rest, physiotherapy, use of anti-inflammatory drugs, and corticosteroid injections are some of the conservative measures most commonly adopted in clinical practice. However, scientific evidence and recommendations for the use of these therapeutic measures are limited. In clinical practice, conservative treatment is unsatisfactory in most cases. Surgical treatment, in turn, also proves to be ineffective, resulting in high rates of complications and recurrences [1,6].

In the last decade, knowing about the reduced tissue regeneration capacity of the Achilles tendon and reducing local pain has shown great interest. Among the various biological therapies that have been proposed and investigated, the use of factors derived from blood, such as platelet-rich plasma (PRP), is increasingly used. PRP is defined as a plasma sample, obtained after blood centrifugation, with a platelet concentration two or more times greater than basal levels or greater than 1.1 × 106/µL. The high concentration of platelets results in the release of several growth factors, such as PDGF, TGF-β1, TGF-β2, PDAF, VEGF, EGF, PDEGF, ECGF, IGF, and bFGF [7,8]. This pool of growth factors has a variety of functions, which include fibroblast mitogenesis, angiogenesis, collagen deposition, and formation of granulation tissue, among others, which are important for accelerating tissue recovery, which is of particular interest in locations with low intrinsic healing potential [9]. A recent meta-analysis concluded that PRP provided an improvement to microfracture in knees and ankles at short-term follow-up. However, the overall low evidence and the paucity of high-level studies indicate that further research is needed to confirm the potential of PRP augmentation to microfracture for the treatment of cartilage lesions [10].

Given these facts, there is growing interest in research aimed at identifying new therapeutic approaches for tendinopathies that offer more effective and safer alternatives. From this perspective, the use of experimental animal models has proved important for a better understanding of the pathophysiology and treatment of tendinopathies. Rats and rabbits are the preferred models, due to their anatomical similarity to the human tendon. Based on these models, a new strategy, the use of biological tissues, is being studied [11]. The application of mononuclear stem cells is supported by two main hypotheses. It is possible that tendon healing may be facilitated by the paracrine effects of stem cell injection, which secrete various growth factors and cytokines essential for the recovery of injured tissues. Another hypothesis suggests that tendon healing may be more appropriate due to the differentiation of stem cells into tenocytes, which are capable of leading to tissue regeneration, i.e., the replacement of damaged fibers with newly formed tissue, identical to the previously healthy tendon [12]. Pre-clinical studies in rabbits indicated accelerated histological and biomechanical recovery in tendons exposed to lacerations and subsequently subjected to intratendinous injections of mononuclear stem cells [13]. 

It is also known that the amniotic membrane, an element that would usually be discarded along with the placenta, is easily obtained after cesarean delivery and has anti-inflammatory and antimicrobial properties, as well as low antigenicity [14,15,16]. The amniotic membrane is characterized by having various growth factors that stimulate chemotaxis, cell proliferation, and differentiation, as well as collagen synthesis, neovascularization, and extracellular matrix deposition. 

Thus, it is possible that mononuclear stem cells and amniotic membranes have therapeutic characteristics, potentially influencing cell differentiation and the formation of a specialized cellular matrix in injured tissues. The use of these elements could be an alternative for the treatment of chronic injuries with fibrotic characteristics, such as tendinopathies injuries whose treatment has a high failure rate. 

Cellular mechanisms involved in tenogenesis remain largely unknown. The signaling pathways that regulate tenogenesis at the translational level are poorly understood, making it essential to carry out studies to identify them. Recently, the AKT-mTOR axis has been shown to be essential for tenogenesis in animal models, specifically for the production of type I collagen in tendons, as well as for tendon differentiation, through translational mechanisms. These findings offer a potential therapeutic target for tendon-repairing strategies [17,18,19,20,21]. 

Interferon-γ is a cytokine that plays multiple roles and is, at the same time, capable of activating immune responses and preventing their exaggerated response, which is often responsible for additional tissue damage. This balance is maintained by complex mechanisms that are still poorly understood [9]. Interferon-γ signaling pathway requires activation of its receptor (IFNGR), with consequent stimulation of JAK/STAT signaling. Activation of the STAT1-PI3K-AKT axis in turn results in activation of the mTOR pathway, while the mTOR/p70S6 kinase cascade promotes translation of the mRNA of effector proteins [22]. 

The aim of this study was therefore to evaluate the effects of using bone marrow stem cells and amniotic membrane in the treatment of Achilles tendinopathy induced by total tendon rupture in rats, compared to the control group, which received saline solution. The concentration, arrangement, and type of collagen present in the lesions were assessed using histomorphometry and immunohistochemistry, as well as the presence of the immunohistochemical markers interferon-γ and AKT-mTOR, signaling pro-healing pathways. Through this research, we aim to contribute to the investigation of new cell therapies for tendinopathy, with great potential for application in clinical practice.

## 2. Materials and Methods

### 2.1. Study Design and Ethics

This is an experimental study, carried out in accordance with the ethical standards and principles of the Brazilian College of Animal Experimentation. This study was approved by the Research Ethics Committee on the Use of Animals of the Pontifical Catholic University of Paraná (CEUA-PUCPR) (Number 01.637). 

#### 2.1.1. Sample

Thirty-seven male Wistar rats, weighing an average of 350 g, were divided into four groups after inducing injury to the Achilles tendon of both hind legs: the Control Group (n = 9), Stem Cell Group (n = 10), Amniotic Membrane Group (n = 10), and Stem Cell and Amniotic Membrane Group (n = 8).

#### 2.1.2. Animal House Conditions

The animals were maintained in the Bioterium of the Paraná Institute of Technology, with a maximum of 4 animals per cage, water and food ad libitum, and 12-h daylight/12-h dark periods, with local temperature and humidity control. They were monitored daily by the researchers, assessing clinical and behavioral signs of post-surgical complications, including parameters of pain or discomfort, visualization of the expansion of the thoracic cavity and respiratory movements per minute, gum coloration (normal: pink; abnormal: bluish) and capillary reperfusion time to estimate blood pressure, and walking and mobility of the paws, as set out in a form specifically designed for the research.

### 2.2. Study Procedures

#### 2.2.1. Anesthesia

The protocol for inducing anesthesia consisted of measuring the animal’s weight on a digital scale and applying intraperitoneal anesthesia with 5% ketamine hydrochloride (Vetanarcolâ, Konig from Brazil Ltd.a., Santana de Parnaíba, Brazil) at a dose of 80 mg/Kg associated with 2% xylazine hydrochloride (Rompunâ, Bayer S.A, Cachoeira Dourada (MG), Brazil) at a dose of 10 mg/Kg. Trichotomy was performed only after the anesthetic plan had been achieved, verified by the pupillary reflex and muscle relaxation.

#### 2.2.2. Induction of Tendon Injury

**Figure 1:**
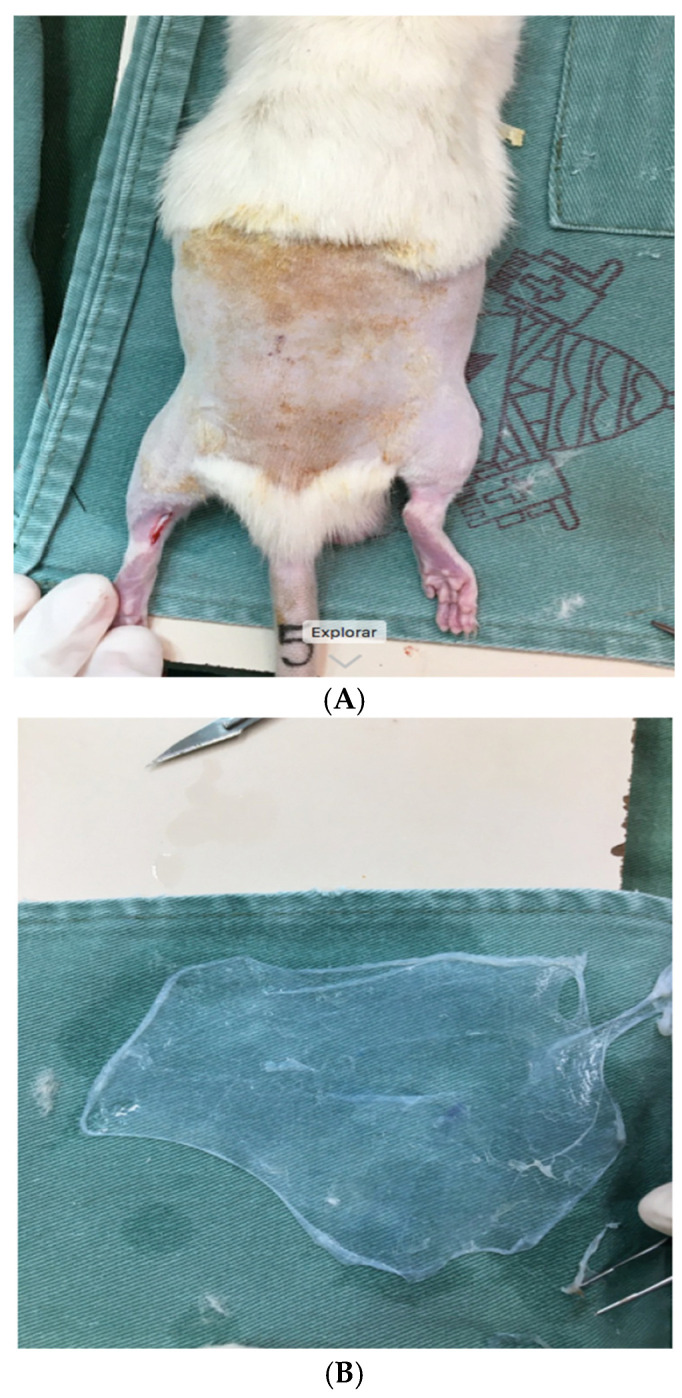
(**A**) Rat being prepared for tenotomy; (**B**) Amniotic membrane immediately before implantation.

**Figure 2:**
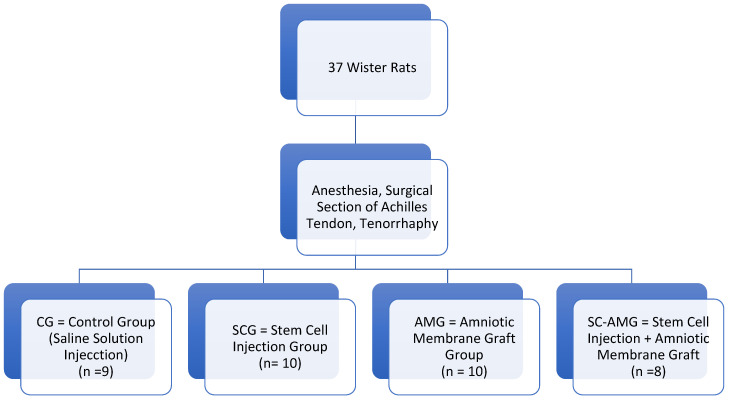
Flowchart—study group distribution.

After anesthesia and trichotomy in the region of the right Achilles tendon, a skin incision was made over the Achilles tendons, identifying and isolating the tendon for its resection, in two identical halves (Figure 1A). They were then sutured using a Kessler stitch and the animals were divided randomly into study groups (Figure 2):

- Control Group (CG): after tenorrhaphy, saline solution was injected into the site;
- Stem Cell Group (SCG): after tenorrhaphy, stem cells were infiltrated into the tendon and around the tendon, in the region where tenorrhaphy was performed, under direct visualization (1 mL, obtained through centrifugation of bone marrow aspirate);
- Amniotic Membrane Group (AMG): after tenorrhaphy, the amniotic membrane was grafted, involving the entire tendon at the site of the injury (Figure 1B);
- Stem Cells and Amniotic Membrane Group (SC-AMG): after tenorrhaphy, stem cells were infiltrated into the site and the amniotic membrane was grafted.

#### 2.2.3. Obtaining and Isolating Bone Marrow Cells—The Mononuclear Fraction

Mononuclear cells were obtained by the puncture–aspiration technique from the iliac crest of the rats, for the isolation of the mononuclear fraction using the density gradient (d = 1.077 g/m^3^) (Ficoll-Hypaque-Sigma, St. Louis, MO, USA) according to Böyum, in Iscove’s modified Dulbecco’s medium (IMDM-GIBCO BRL) supplemented with 1% antibiotic (penicillin and streptomycin) and 20% buffer solution. 

To do this, the material collected from each rat was placed in a sterile 15 mL plastic centrifuge tube and the contents were topped up to 12 mL with IMDM (Iscove’s Modified Dulbecco’s Media) culture medium, supplemented with 4% buffer solution and 1% antibiotic (penicillin and streptomycin) and homogenized. 

In another 15 mL plastic tube, 3 mL of density gradient separation solution (d = 1.077 Ficoll-Hypaque) was added, followed by the homogenate containing the animal’s bone marrow and IMDM culture medium, carefully so that it did not mix. The tube was then taken to the centrifuge and subjected to 1400 revolutions per minute (rpm) for 40 min at 22 °C. Afterward, it was taken to flow again and the ring formed between the medium and the gradient was removed.

The homogenate obtained (where the mononuclear stem cells were located) was then removed and transferred to another 15 mL plastic tube. This new tube was topped up to 15 mL with IMDM medium and centrifuged again at 1500 rpm for 10 min at 22 °C. The tube was then removed from the centrifuge and the supernatant was quickly discarded. The precipitate found at the bottom of the tube after this step corresponded to the mononuclear stem cells used in the study. The previous step was repeated and after adding 13 mL of medium to the tube, the precipitate was resuspended and centrifuged again at 1500 rpm for a further 10 min at 22 °C. After this phase, the supernatant was discarded, 3 mL of medium was added to the tube, and the cells were resuspended for counting. This count was carried out in a Neubauer chamber and read using an Olympusa CX31 optical microscope with a 40× objective.

#### 2.2.4. Obtaining the Amniotic Membrane 

Amniotic membranes were obtained from the placentas of two parturients (36 and 40 weeks of gestational age, respectively), from the Victor Ferreira do Amaral Maternity Hospital, through a project approved by CEUA-PUCPR. The use of placentas to obtain amniotic membranes was subject to an authorization form approved by the hospital and a Free and Informed Consent Form signed by the patients. 

#### 2.2.5. Amniotic Matrix Decellularization

The Amniotic Matrix was decellularized using an aseptic technique in a BioSAFE class II biological safety cabinet (Veco^®^, Campinas, Brazil). For this process, the membranes were removed from the phosphate-buffered saline (PBS) pH 7.2 (Gibco^®^, São Paulo, Brazil) and treated with 0.01% SDS (sodium duodecyl sulfate) and 0.01% SD (sodium deoxycholate) for 24 h at 37 °C, using a mechanical shaker (Shaker Table 109 M, Novatécnica^@^, Piracicaba, Brazil). They were then preserved in PBS at 4 °C, according to the methodology described by Hopper, Woodhouse, and Semple [23].

#### 2.2.6. Preparation of the Decellularized Human Amniotic Matrix

Fresh amniotic membranes obtained after cesarean deliveries were from donors whose serologies were negative for Human Immunodeficiency Virus (HIV), Hepatitis B, Hepatitis C, and syphilis. The blood clots were immediately removed from the placenta with phosphate-buffered saline (PBS) containing 100 U/mL penicillin and 100 mg/mL streptomycin. A decellularized human amniotic matrix was prepared using the method recommended by Riau et al. [24]. Amniotic epithelial cells were removed from the human amniotic membrane using sodium dodecyl sulfate in PBS and incubated at a rotation speed of 100 rpm at 37 °C for 24 h [24].

#### 2.2.7. Preparing Tissue Samples for Histological and Immunohistochemical Evaluation

Six weeks after the surgical procedure, the animals were euthanized to remove the Achilles tendons. Tissue samples of Achilles tendons were obtained from the injured sites and evaluated using a histological score and immunohistochemical markers (Interferon-γ, AKT and mTOR).

##### Histomorphometric Evaluation

The morphometric evaluation took into account cellularity, the presence of vessels, and collagen fibers. Histological sections were obtained exactly from the section and suture site, placed on slides, and stained with hematoxicillin eosin solution for nuclear and extracellular matrix characterization and Picrosirus red specifically for type I and III collagen fibers. The density of collagen fibers was estimated using the following formula: % of reticular fibers (type III collagen) = sum of areas with the same density (stained with Picrosirius red) in the 10 × field/cross-sectional area of the tendon ×100.

**Figure 3:**
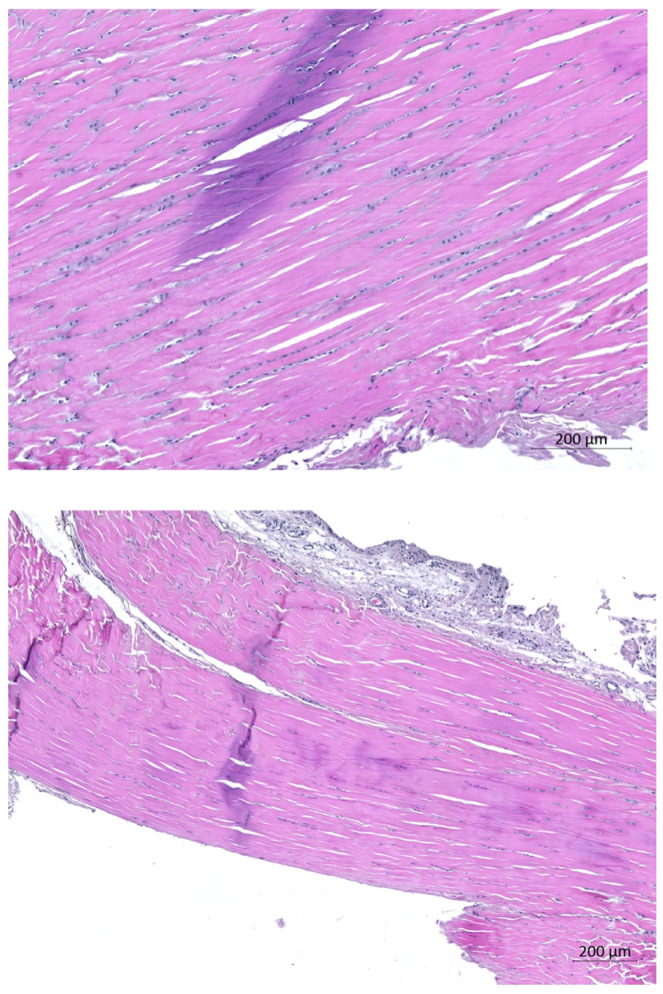
Slides showing the section of the Achilles tendon of one of the rats from the Stem Cell–Amniotic Membrane Group prepared for histological evaluation.

Histological evaluation was carried out by analyzing 6 parameters, according to a score previously proposed, ranging from 1 (significant alterations) to 4 (normal appearance) [25]. The linearity of the collagen fiber structure, tenocyte shape, tenocyte density, signs of inflammation, hemorrhage, and epitendon thickness were evaluated. The higher the scores, the closer the samples were to normal tissue. Scores were assigned by a single expert, who was an independent observer and blinded to the study groups. Figure 3 shows two samples prepared for histological evaluation.

##### Immunohistochemical Evaluation

Achilles tendons were placed in 5-micrometer histological sections on slides and stained, making comparisons between sections from the study and control groups. Sections were also taken for immunohistochemical procedures using antibodies that identified interferon-γ and the presence of AKT-mTOR markers. These markers belong to the same signaling pathway, involved in a pro-healing state, and were expressed according to the density in which they were found in the tissues observed.

Immunohistochemical analysis was carried out using previously validated antibodies to identify endothelial cells, endothelial, neural, and vascular growth factors, myotendinous junction, type I collagen, type 3 collagen, and stem cell transcription factor. Staining was performed against collagen I (1:500, ABcam^®^, Cambridge, UK), III (1:800, ABcam^®^, Cambridge, UK), versican (1:500, Millipore^®^, Burlington, VT, USA), and agrecan (1:1000, Millipore^®^, Burlington, VT, USA). Neovascularization was assessed using RECA-1 (ABcam^®^, Cambridge, UK, 1:50), and the number of vessels was counted using 40× magnification microscopic fields.

The AKT-mTOR marker from the cloud-clone brand at a dilution of 1:100 Ph6 cod./clone PAA 806Hu01 and AKT3 from the Abcam^®^ (Cambridge, UK), clone 66C12471 at a dilution of 1:400 were used.

Interferon-γ marker from the Abcam^®^ (Cambridge, UK), clone ab218426, was used in a 1:100 dilution.

### 2.3. Statistical Analysis

Quantitative variables evaluated in the study were described by means, standard deviations, medians, minimum values, and maximum values. Qualitative variables were described by frequencies and percentages. The one-way analysis of variance (ANOVA) model or the non-parametric Kruskal–Wallis test and Dunn’s post-hoc test were used to compare groups in relation to the quantitative variables. The correlation between two quantitative variables was analyzed by estimating Spearman’s correlation coefficients and assessing their significance. The normality of continuous variables was assessed using the Shapiro–Wilk test. With regard to qualitative variables, groups were compared using Fisher’s Exact Test or the Chi-squared test. Values of *p* < 0.05 indicated statistical significance. For multiple comparisons, *p*-values were corrected by Bonferroni. Data were analyzed using the IBM SPSS Statistics v.20 software.

## 3. Results

Thirty-seven male Wistar rats weighing an average of 350 g were randomly divided into four groups after inducing injury to the Achilles tendon of both hind legs: 9 rats in the CG (saline solution), 10 rats in the SCG, 10 rats in the AMG, and 8 rats in the SC-AMG.

Morphometric histological evaluation of Achilles tendons of the samples were studied according to the following parameters: linearity of collagen fiber structure, tenocyte shape, tenocyte density, inflammation, hemorrhage, and epitendon thickness. Histological scores ranging from 12 to 17 were observed in the CG, from 10 to 17 in the SCG, from 12 to 14 in the AMG, and from 9 to 14 in the SC-AM Group. Mean histological scores were between 12.6 (SC-MAG) and 14.2 (GC), with medians between 13.5 and 14. There were no statistically significant differences between the groups (Table 1).

The average percentage of Interferon-γ, observed by immunohistochemical analysis among all the animals in the four study groups, is shown in Table 2. Due to the technique used for paraffin embedding, some samples were lost, with a reduction in the number of samples.

**Scheme 1:**
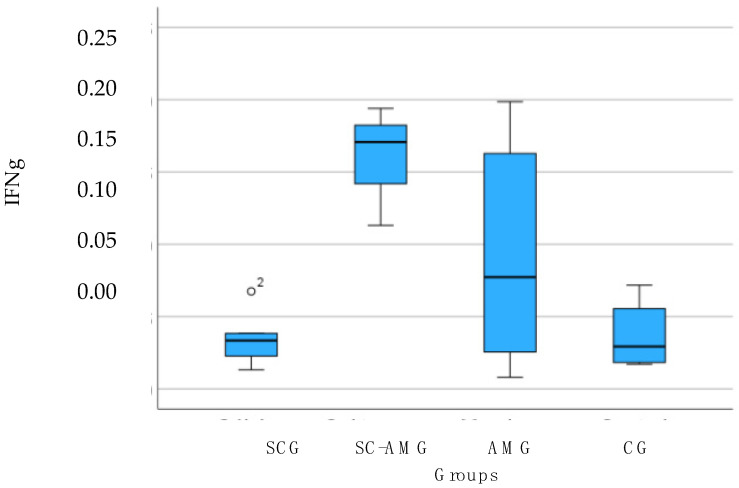
Interferon-γ expression in medians, 1st and 3rd quartiles, minimum and maximum values—comparison between the control (saline solution), stem cells, amniotic membrane, and stem cells + amniotic membrane groups (n = 31). Legend: CG = Control Group; SCG = Stem Cells Group; AMG = Amniotic Membrane Group; SC-AMG = Stem Cells and Amniotic Membrane Group.

There was a significant difference between the groups in terms of Interferon-γ expression, so comparisons were made between the groups two by two, as shown in Table 3. Differences showed statistical significance in the comparison between the SCG and the SC-AMG, with a higher percentage of Interferon-γ expression in the latter group. Scheme 1 illustrates these results according to medians and interquartile ranges.

Expression of the AKT-2 and mTOR markers in the different study groups are described in Table 4 and Table 5. No significant differences were found between the groups.

**Figure 4:**
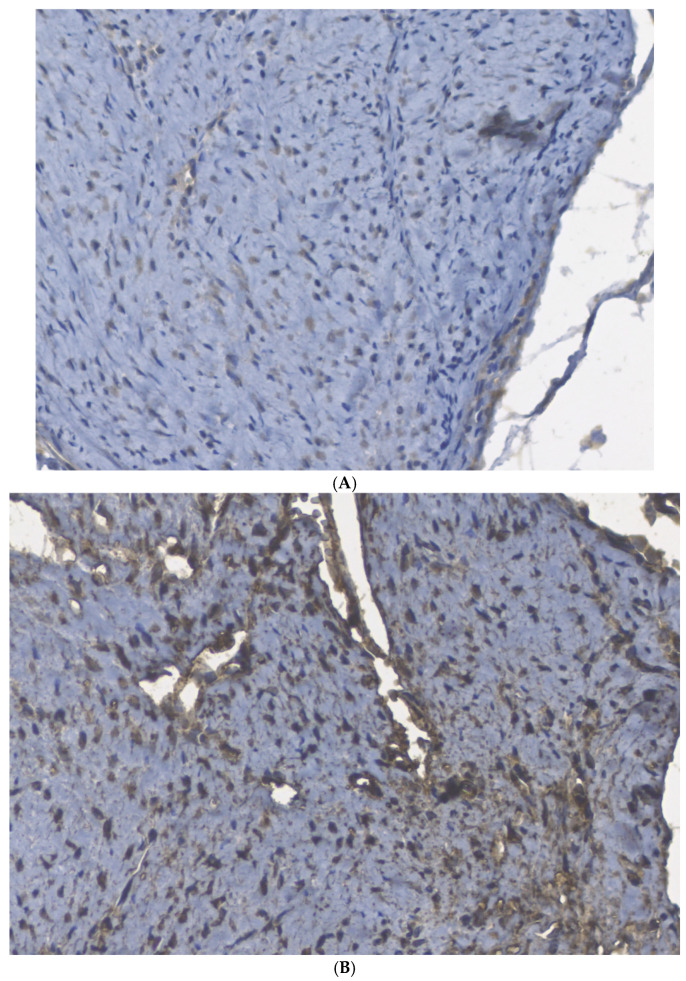

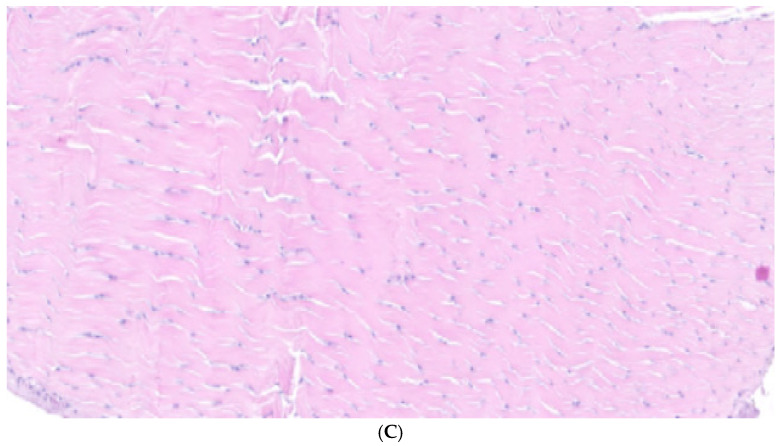
Slides showing a section of the Achilles tendon of one of the rats from the Stem Cell–Amniotic Membrane Group: (**A**) Immunohistochemical evaluation of the AKT marker in the Achilles tendon; (**B**) Immunohistochemical evaluation of the mTOR marker in the Achilles tendon; (**C**) Hematoxylin–eosin evaluation in the Achilles tendon.

Figure 4 shows the images of scanned slides of the Achilles tendon of one of the rats in the sample, with the immunohistochemical evaluation of the AKT-mTOR markers and hematoxylin–eosin staining, respectively.

## 4. Discussion

This experimental study aimed to verify the impact of treatment with bone marrow stem cells, amniotic membranes, and a combination of both after surgical sectioning of Achilles tendons in rats with the objective of assessing various parameters such as histological evaluation of the tissues, the density of Type I and Type III collagen (potentially involved in the “quality of healing”), and the expression of regulators of the inflammatory process, such as interferon-γ and the signaling pathways involved in the translation of effector proteins (AKT-mTOR). 

No significant differences were observed between the CG and the Intervention Groups in terms of the morphometric histology of the tendons (histological scores) nor in terms of the density of Type I (which predominated in all groups) and Type III collagen. The main finding was a significantly higher expression of interferon-γ in the SC-AMG when compared to the SCG (*p* = 0.011), with borderline values for the comparison between the CG and SC-AMG (*p* = 0.081). These findings suggest that the combined treatment of Stem Cells with an Amniotic Membrane may have favored the expression of the cytokine interferon-γ, involved in the inflammatory process resulting from tendon injury.

One of the hypotheses supporting the efficacy of stem cell treatments for tendinopathies suggests that tendon healing may be facilitated by the paracrine effects of injecting stem cells, which secrete various growth factors and cytokines, essential for the recovery of injured tendons [11,12]. Recently, the therapeutic potential of mesenchymal stromal cells derived from the human amniotic membrane has been directed toward both soluble factors and extracellular vesicles, with an abundance of factors and RNAs released by these cells. The molecules identified have been shown to reduce inflammation and degeneration in injured osteoarthritic joints and tendons, according to preliminary results from in vitro and preclinical studies [26]. Similar results were reported in a skin lesion rat model, which found that the application of stem cells associated with an amniotic membrane accelerated the healing phase, probably owing to their anti-inflammatory effect that favored the early formation of collagen and elastic fibers [27,28]. 

However, the present study was unable to show a positive correlation between interferon-γ expression and improved histological scores or increased Type I collagen density. And, when all groups were assessed, there was a negative correlation, with a low degree of association (r = −0.42, *p* = 0.018) between interferon-γ expression and Type I Collagen density. These results are possibly due to the small sample size in each of the groups, both the CG and the Intervention Groups. The grouping of cases in the context of small samples possibly interfered with the results of this analysis and it would have been more appropriate to analyze each intervention group separately, with larger samples.

It is important to note that the role of interferon-γ in modulating the inflammatory reaction and tendon healing has complex mechanisms that are not yet fully understood. A recent experimental study that sought to evaluate Achilles tendon repair in rats after splenectomy found that in the group submitted to splenectomy, there was a reduction in pro-inflammatory cytokines such as interleukin-1β, tumor necrosis factor-α, and interferon-γ, associated with an improvement in the tensile strength of the tendons examined after four weeks. For these authors, very little is known about the interaction between the immune system and the recovery of injured tendons but their findings suggest that splenectomy positively influenced the healing of Achilles tendons through changes in the pro-inflammatory and anti-inflammatory environment related to cytokine secretion [29].

The expression of AKT-mTOR pathways has been the subject of research since it has been shown that this axis is essential for tenogenesis in animal models, specifically for the production of type I collagen in tendons, as well as for tendon differentiation, through translational mechanisms [17,18,19,20,21,22]. However, the present study failed to establish any relationship between AKT-mTOR expression in the different control and intervention groups, either in terms of histological score changes or in the assessment of type I collagen density. The small sample size of each group possibly had an impact on the analysis of the results obtained. 

Tendinopathies are among the musculoskeletal disorders that most frequently affect the locomotor system. They are characterized by pain and loss of physical function, which often result in an increased risk of developing other chronic diseases and health problems, including obesity, a sedentary lifestyle, and a further significant reduction in mental health [26].

In the United States of America, it is believed that one in two adults suffer the consequences of musculoskeletal disorders, a prevalence comparable to heart disease and chronic respiratory disease combined [30]. Around 33 million musculoskeletal injuries are reported every year, of which 50% are tendon and ligament injuries. Among these, the Achilles tendon is one of the most commonly involved, due to excessive and repetitive use [30]. Although the prevalence of tendinopathies increases with age, these conditions are not exclusive to the elderly population. Achilles tendinopathy is not restricted to physically active young people and adults, as it can also occur in inactive or moderately active individuals [7,31,32].

The well functioning of the musculoskeletal system is critical for human beings, as it guarantees mobility, dexterity, and the ability to work and actively participate in all aspects of life in society and is essential for functional, social, and economic independence. Musculoskeletal disorders currently represent one of the main reasons for the loss of workforce and productivity, the need for early retirement, and a reduction in society’s financial security to a greater extent than contagious diseases. It is estimated that the costs related to these injuries exceeded USD 200 billion in the United States in 2011 [30]. In this scenario, Achilles tendon injuries are particularly devastating because, unlike some types of tissue, these tendons are poorly vascularized structures and, instead of the formation of tissue homologous to the native healthy tissue, there is the formation of a fibrous scar tissue, which makes it mechanically weaker and increases the risk of further complications [11].

Various types of treatment for Achilles tendinopathy have been proposed, but their results have not been satisfactory. The main problem associated with conservative treatments is that, in most cases, they only aim to reduce nociception but do not seek to intervene in the pathophysiological processes of the disease itself. In cases that are refractory to conservative approaches (around 30% of patients), surgical treatment is necessary, with low success rates. To overcome these obstacles, specialists have shifted their focus to the use of orthobiologics as new therapeutic tools [11]. The treatment of Achilles tendinopathy with orthobiologics aims to restore the original properties of the tendon tissue and reduce the susceptibility to secondary injuries and further damage. Current optimal therapeutic management aims at regeneration rather than tendon reconstruction. Tendon regeneration requires not only the balance of the extracellular matrix but also the presence of cellular components, justifying the growing interest in cell therapies [7,12,32,33].

Although the participation of several orchestrated signaling pathways in tendon healing has been demonstrated, their mechanisms of regulation and functioning are complex and not yet fully understood. Several studies have proposed therapeutic approaches using stem cells and tissue engineering in an attempt to reverse the degeneration process of tendinopathies and promote tissue regeneration [34].

The use of different types of stem cells based on their paracrine effects and cell differentiation potential has been extensively investigated [8,9,11,12,35,36,37,38]. However, despite great progress in identifying the molecular regulatory factors and cells involved in tendon healing, no major therapeutic breakthroughs toward significantly improving the repair of injured tendons have yet been achieved. Several knowledge gaps and challenging problems present themselves with cell therapy and tissue transplantation. Obtaining stem cells can raise ethical questions while transplanting allogeneic cells can lead to immune reactions. This problem can be overcome with the use of autologous cells, but obtaining these can be related to significant complications or morbidities for the donor. Another difficulty is the phenotypic alteration of the cells and the loss of the desired characteristics and properties during their expansion in vitro [39]. It should also be noted that stem cells can cause serious damage, despite their healing potential. These include the risk of infections due to bacterial contamination and even the development of tumors, such as kidney and brain tumors observed in patients who received stem cell therapy to treat kidney failure and ischemic brain injury, respectively. In the case of stem cell administration for the treatment of tendinopathies, no serious adverse effects have been reported to date, but the risk of ectopic bone formation and tumor development in some circumstances cannot be ruled out [11].

Besides the relatively small sample, other limitations of this study should be considered. In the experimental model carried out, Achilles tendon injury was obtained through surgical sectioning of the tendon. This type of injury certainly differs from the degenerative lesions found after repeated stress and overload, without total rupture of the tendon. Several methods of inducing Achilles tendon injury have been applied in animal models, including repetitive contractions of the triceps sural (mechanical overload), the application of inflammatory cytokines and corticosteroids, or different types of trauma, penetrating or blunt [3,6,40]. It should also be noted that animal models may not faithfully reproduce the mechanisms of tendinopathy and tendon repair in humans. However, rats, the animals used in this study, represent a good experimental model of tendinopathies. The size of their tendons allows surgical or percutaneous procedures to be carried out and adequate amounts of tissue can be collected for laboratory analysis.

In future studies, strategies will be considered to minimize these limitations and strengthen this research, such as making larger samples available, studying different injury mechanisms, and expanding the anatomical and functional parameters of tendon recovery.

In summary, in recent years, a great deal of information has been produced on the anatomy, biomechanical properties of normal and healing Achilles tendons, the pathophysiology of tendinopathies, and tissue repair processes. However, there are still many knowledge gaps to be clarified. In this scenario, well-designed experimental studies are important to resolve fundamental questions and propose consistent treatment modalities, while large-scale clinical trials being essential to identify the ideal therapeutic measures, with the greatest evidence of benefit and the lowest risks to patients. Future studies should focus particularly on the healing of the Achilles tendon and seek to investigate not only its composition but also its structural, mechanical, and functional properties. These aspects are fundamental for proposing new therapies that can effectively translate into satisfactory clinical outcomes. Research into biologics, including cell therapies, therapeutic use of cytokines and growth factors, genetic modifications, and tissue engineering are currently realistic perspectives for the treatment of Achilles tendinopathies, and efforts to understand their complex mechanisms are necessary in order to enable new effective and safe therapeutic options.

In conclusion, the isolated use of mononuclear stem cells, amniotic membranes, or the combined use of mononuclear stem cells and amniotic membrane, when compared to the control group, did not produce significant changes in relation to the concentration, arrangement, or type of collagen, nor in relation to the number of inflammatory cells and fibroblasts, assessed by means of the histological score of the Achilles’ tendons sectioned in rats. In the immunohistochemical evaluation, although no significant difference was observed in the expression of the AKT-2 and mTOR markers, in the groups in which the association of mononuclear stem cells and the amniotic membrane was applied, an increase in the expression of Interferon-γ was observed when compared to the other groups. These findings suggest that the use of biological therapies may favor healing in tendinopathy processes, but further studies are needed to better clarify these cellular pathways.

## Figures and Tables

**Table 1:** Morphometric histological evaluation of the Achilles tendons of Wistar rats studied according to the histological score—a comparison between groups.

Group	n	Mean	Standard Deviation	Median	Minimum	Maximum	*p* *
CG	9	14.2	1.3	14.0	12.0	17.0	0.22
SCG	10	13.6	1.8	13.5	10.0	14.0	
AMG	10	13.6	0.7	14.0	12.0	14.0	
SC-AMG	8	12.6	1.8	13.5	9.0	14.0	

Legend: CG = Control Group; SCG = Stem Cells Group; AMG = Amniotic Membrane Group; SC-AMG = Stem Cells and Amniotic Membrane Group. * Kruskal–Wallis Non-Parametric Test.

**Table 2:** Expression of Interferon-γ in the Achilles tendons of the Wistar rats studied—a comparison between groups.

Group	n	Mean	Standard-Deviation	Median	Minimum	Maximum	*p* *
CG	7	0.05	0.05	0.04	0.02	0.15	0.014
SCG	9	0.04	0.03	0.03	0.01	0.10	
AMG	8	0.09	0.07	0.08	0.01	0.20	
SC-AMG	7	0.17	0.09	0.17	0.06	0.32	

Legend: CG = Control Group; SCG = Stem Cells Group; AMG = Amniotic Membrane Group; SC-AMG = Stem Cells and Amniotic Membrane Group. * Kruskal–Wallis Non-Parametric Test.

**Table 3:** Expression of Interferon-γ in the Achilles tendons of Wistar rats studied—two-way comparison between groups (n = 31).

Compared Groups	Compared N	*p*
CG × SCG	7 × 9	1.0
CG × AMG	7 × 8	1.0
CG × SC-AMG	7 × 7	0.081
SCG × AM	9 × 8	1.0
SCG × SC-AMG	9 × 7	0.011
AMG × SC-AMG	8 × 7	0.45

Legend: CG = Control Group; SCG = Stem Cells Group; AMG = Amniotic Membrane Group; SC-AMG = Stem Cells and Amniotic Membrane Group—Dunn’s test.

**Table 4:** AKT-2 expression in the Achilles tendons of the Wistar rats studied—a comparison between groups.

Group	n	Mean	Standard-Deviation	Median	Minimum	Maximum	*p* *
CG	8	381.1	631.8	65.9	44.6	1766.9	0.643
SCG	7	307.8	658.7	54.0	32.1	1800.8	
AMG	8	890.7	1656.9	57.1	33.2	4517.1	
SC-AMG	7	54.3	15.2	49.9	34.3	74.8	

CG = Control Group; SCG = Stem Cells Group; AMG = Amniotic Membrane Group; SC-AMG = Stem Cells and Amniotic Membrane Group * Kruskal–Wallis Non-Parametric Test.

**Table 5:** Expression of mTOR in the Achilles tendons of Wistar rats studied—a comparison between groups.

Group	n	Mean	Standard-Deviation	Median	Minimum	Maximum	*p* *
CG	7	60.1	22.4	56.4	37.2	107.6	0.112
SCG	7	1594.7	2906.2	48.0	34.8	7541.7	
AMG	9	1138.8	3005.9	58.2	42.2	9133.4	
SC-AMG	6	45.0	10.0	47.7	27.5	56.3	

Legend: CG = Control Group; SCG = Stem Cells Group; AMG = Amniotic Membrane Group; SC-AMG = Stem Cells and Amniotic Membrane Group. * Kruskal–Wallis Non-Parametric Test.

## Data Availability

Data is contained within the article. The original contributions presented in this study are included in the article. Further inquiries can be directed to the corresponding author(s).
